# Supplementary material for: Extensive sheep grazing is associated with trends in steppe birds in Spain: recommendations for the Common Agricultural Policy
Source: PeerJ. 2022 Feb 28;10:e12870. doi: 10.7717/peerj.12870 (PMC8893029; doi:10.7717/peerj.12870)
Supplement: Supplemental Information 2 [file peerj-10-12870-s002.docx]

Table S1: List of species included in the Farmland Bird Index for Spain. English and Latin names, and trend in Spain for the period 1998-2018 are provided.

| Common name | Latin name | Trend in Spain |
| --- | --- | --- |
| European Bee-eater | *Merops apiaster* | Moderate decline |
| Eurasian Hoopoe | *Upupa epops* | Stable |
| Woodchat Shrike | *Lanius senator* | Moderate decline |
| Red-backed Shrike | *Lanius collurio* | Moderate decline |
| Eurasian Skylark | *Alauda arvensis* | Moderate decline |
| Calandra Lark | *Melanocorypha calandra* | Moderate decline |
| Eurasian Kestrel | *Falco tinnunculus* | Moderate decline |
| White Stork | *Ciconia ciconia* | Moderate increase |
| Zitting Cisticola | *Cisticola juncidis* | Moderate increase |
| Common Quail | *Coturnix coturnix* | Moderate decline |
| Crested Lark | *Galerida cristata* | Moderate decline |
| Western Black-eared Wheatear | *Oenanthe hispanica* | Moderate decline |
| Yellowhammer | *Emberiza citrinella* | Moderate decline |
| Cirl Bunting | *Emberiza cirlus* | Moderate decline |
| Corn Bunting | *Emberiza calandra* | Moderate decline |
| Spotless Starling | *Sturnus unicolor* | Moderate increase |
| European Starling | *Sturnus vulgaris* | Stable |
| Barn Swallow | *Hirundo rustica* | Moderate decline |
| Rock Sparrow | *Petronia petronia* | Moderate increase |
| House Sparrow | *Passer domesticus* | Moderate decline |
| Eurasian Tree Sparrow | *Passer montanus* | Moderate decline |
| Spanish Sparrow | *Passer hispaniolensis* | Moderate increase |
| Eurasian Jackdaw | *Corvus monedula* | Moderate decline |
| European Goldfinch | *Carduelis carduelis* | Stable |
| Little Owl | *Athene noctua* | Moderate decline |
| Eurasian Linnet | *Linaria cannabina* | Stable |
| Red-legged Partridge | *Alectoris rufa* | Moderate decline |
| European Serin | *Serinus serinus* | Moderate decline |
| Little Bustard | *Tetrax tetrax* | Moderate decline |
| Greater Short-toed Lark | *Calandrella brachydactyla* | Moderate increase |
| European Turtle-Dove | *Streptopelia turtur* | Moderate decline |
| European Magpie | *Pica pica* | Moderate decline |
| European Greenfinch | *Chloris chloris* | Moderate increase |

Table S2: List of species included in the Steppe Bird Index for Spain. English and Latin names, and trend in Spain for the period 1998-2018 are provided.

| Common name | Latin name | Trend in Spain |
| --- | --- | --- |
| Eurasian Thick-nee | *Burhinus oedicnemus* | Moderate decline |
| Woodchat Shrike | *Lanius senator* | Moderate decline |
| Iberian Gray Shrike | *Lanius meridionalis* | Moderate decline |
| Eurasian Skylark | *Alauda arvensis* | Moderate decline |
| Wood Lark | *Lullula arborea* | Moderate increase |
| Tawny Pipit | *Anthus campestris* | Stable |
| Thekla’s Lark | *Galerida theklae* | Moderate increase |
| Northern Wheatear | *Oenanthe oenanthe* | Moderate decline |
| Western Black-eared Wheatear | *Oenanthe hispanica* | Moderate decline |
| Spectacled Warbler | *Curruca conspicillata* | Stable |
| Corn Bunting | *Emberiza calandra* | Moderate decline |
| Pin-tailed Sandgrouse | *Pterocles alchata* | Moderate increase |
| Black-bellied Sandgrouse | *Pterocles orientalis* | Moderate decline |
| Little Owl | *Athene noctua* | Moderate decline |
| Eurasian Linnet | *Linaria cannabina* | Stable |
| Red-legged Partridge | *Alectoris rufa* | Moderate decline |
| Little Bustard | *Tetrax tetrax* | Moderate decline |
| European Stonechat | *Saxicola rubicola* | Moderate decline |
| Greater Short-toed Lark | *Calandrella brachydactyla* | Moderate increase |
| Mediterranean Short-toed Lark | *Alaudala rufescens* | Uncertain |

**Table S3**. Estimates of the linear regressions assessing: i) the annual change rate of sheep numbers over the period 1992-2020, ii) the relationship between bird population trends (FBI, SBI) and sheep numbers over 1998 and 2018 (1998 used as reference value), and iii) the relationship between the Dupont’s lark trend and sheep numbers between 2004 (reference value) and 2015. Numbers of degrees of freedom and R^2^ for each are included in the text.

| **Model** |  | Estimate | Std. Error | z value | P |
| --- | --- | --- | --- | --- | --- |
| Sheep numbers | (Intercept) | 805.13 | 69.311 | 11.62 | <0.001 |
|  | Year | -0.39 | 0.035 | -11.21 | <0.001 |
| FBI | (Intercept) | -1.22 | 1.343 | -0.91 | 0.374 |
|  | Sheep trend (1998) | 0.38 | 0.060 | 6.31 | <0.001 |
| SBI | (Intercept) | 0.31 | 1.392 | 0.22 | 0.827 |
|  | Sheep trend (1998) | 0.33 | 0.063 | 5.23 | <0.001 |
| Dupont’s lark | (Intercept) | -14.87 | 6.801 | -2.186 | 0.057 |
|  | Dupont’s trend (2004) | 0.682 | 0.324 | 2.107 | 0.064 |

Figure S1. Map showing all 10x10 UTM squares surveyed under the Spanish Common Bird Monitoring Program (SACRE) between 1998 and 2018.
